# A prospective observational study of iron isomaltoside in haemodialysis patients with chronic kidney disease treated for iron deficiency (DINO)

**DOI:** 10.1186/s12882-018-1159-z

**Published:** 2019-01-10

**Authors:** Ashraf I. Mikhail, Staffan Schön, Sylvia Simon, Christopher Brown, Jörgen B. A. Hegbrant, Gert Jensen, Jason Moore, Lennart D. I. Lundberg

**Affiliations:** 1 Renal Unit, Morriston Hospital, Swansea, Wales UK; 2 Diaverum AB, Lund, Sweden; 3 Medical Department, Pharmacosmos A/S, Holbaek, Denmark; 4 Department of Molecular and Clinical Medicine/Nephrology, The Institute of Medicine, Sahlgrenska Academy, University of Gothenburg, Gothenburg, Sweden; 5 Renal Unit, Royal Devon and Exeter Hospital NHS Foundation Trust, Exeter, UK; 6 Dialysis Department, Renal Medicine, Norrland University Hospital, Umeå, Sweden

**Keywords:** Chronic kidney disease, Haemodialysis, Iron deficiency, Iron isomaltoside, Haemoglobin

## Abstract

**Background:**

Iron deficiency is frequent in haemodialysis (HD) patients with chronic kidney disease (CKD), and intravenous iron is an established therapy for these patients. This study assessed treatment routine, effectiveness, and safety of iron isomaltoside (IIM) 5% (Diafer®) in a HD cohort.

**Methods:**

This prospective observational study included 198 HD patients converted from iron sucrose (IS) and treated with IIM according to product label and clinical routine. Data for IIM were compared to historic data for IS in 3-month intervals. The primary endpoint was to show non-inferiority for IIM versus IS in haemoglobin (Hb) maintenance.

**Results:**

Most patients (> 60%) followed a fixed low-dose iron treatment protocol. Three minutes were required for preparation and administration of IIM. Erythropoiesis-stimulating agent (ESA) was used in > 80% of patients during both IIM and IS phases. The maintenance of Hb was similar with both iron drugs; the mean Hb level was 11 g/dL, and the mean change of 0.3 g/dL (95% confidence interval: 0.1, 0.5) for IIM 0–3 months compared to IS demonstrated non-inferiority. Nine adverse drug reactions were reported in 2% of patients administered IIM. All patients had uneventful recoveries. The frequency of metallic taste was higher with IS compared to IIM (34% versus 0.5%, *p* < 0.0001).

**Conclusions:**

IIM is effective and well tolerated by CKD patients on HD. IIM was non-inferior to IS in maintenance of Hb, and had similar ESA requirements. The fast-push injection of IIM may enable logistical benefits in clinical practice, and the low frequency of metallic taste contributes to patient convenience.

**Trial registration:**

ClinicalTrials.gov identifier NCT02301026, study registered November 25, 2014.

## Background

Iron deficiency is common in patients with chronic kidney disease (CKD), and intravenous (IV) iron is the treatment of choice for those on haemodialysis (HD) [1, 2]. HD patients with CKD may suffer from iron deficiency due to continuous blood losses, treatment with erythropoiesis-stimulating agents (ESAs), impaired absorption of iron by medications such as gastric acid inhibitors and phosphate binders, or impaired absorption of iron and its release from iron stores in inflammation [1]. The amount of iron lost in these patients as a result from blood loss in the HD procedure, regular blood sampling, and occult intestinal bleeding due to uremic enteropathy, is estimated to range from 0.5 to 2.8 g of iron per year depending on if only dialysis-based or if other sources of blood loss are also considered, including the influence of vascular access and comorbidities [3, 4]. Iron deficiency, together with other factors such as insufficient erythropoietin production by the kidneys and hyporesponsiveness to erythropoietin, cause anaemia in these patients [5, 6].

IV iron and ESAs are the cornerstones of anaemia management in CKD patients receiving dialysis [1]. A low-dose high-frequency IV iron treatment regimen is recommended for adult HD patients (> 2 infusions, for each infusion 100–200 mg iron) [2]. Much evidence indicates that adequate iron supply is necessary to achieve optimal responses to ESAs [1], and thereby potentially avoid the risk of cardiovascular and fatal events associated with ESAs [7]. Appropriate treatment with IV iron can allow a decrease in ESA dose in CKD patients [5], and ESA-sparing IV iron therapies may reduce the costs of anaemia management [8].

When prescribing IV iron treatment it is recommended to first balance the potential benefits of reducing blood transfusions, ESA use, and anaemia-related symptoms, against the risk of adverse events associated with parenteral iron preparations [1]. Of particular concerns regarding the risk of adverse events are hypersensitivity reactions and cellular toxicity associated with catalytic/labile iron. Newer formulations of IV iron have, however, demonstrated a low frequency of serious and severe hypersensitivity reactions in several clinical trials [9]. Furthermore, data indicate that IV irons that are more stable with a lower labile iron release and that cause less cell iron uptake have a reduced likelihood of inducing cellular damage [10, 11].

Iron isomaltoside (IIM) 5% (Diafer®) is a low-dose high-frequency IV iron indicated for the treatment of iron deficiency in dialysis CKD patients. It consists of iron and a carbohydrate moiety where the iron is tightly bound in a matrix structure, which minimises the release of labile iron and lowers the risk of iron toxicity [12, 13]. The tight iron binding also allows for fast-push injection. In a previous randomised trial, IIM was found to have comparative efficacy to iron sucrose (IS), and a good safety profile similar to the short-term safety of IS in CKD patients on HD [14]. The use of IS in the HD setting is currently widespread, and this IV iron preparation has a well-established efficacy and short-term safety profile [15]. Concerning the long-term safety of IS, this remains to be investigated in upcoming studies such as the Proactive IV Iron Therapy in Dialysis Patients (PIVOTAL) trial that aims to evaluate all-cause mortality and non-fatal cardiovascular events for IS therapy in a HD population with 2–4 years follow-up per patient [16]. The safety profile of IS over a longer duration of treatment may differ compared to IIM because IS has been shown to release more labile iron [12, 17].

The DINO (DIafer NOn-interventional) study was established to determine the treatment routine, effectiveness, and safety of IIM in a contemporary HD cohort with CKD converted from IS in clinical practice. The treatment outcomes were compared with historic data on IS therapy in the patients in 3-month intervals. The primary endpoint was to demonstrate non-inferiority for IIM versus IS in the maintenance of haemoglobin (Hb) levels over the first 3 months.

## Methods

### Study design and population

**Fig. 1:**
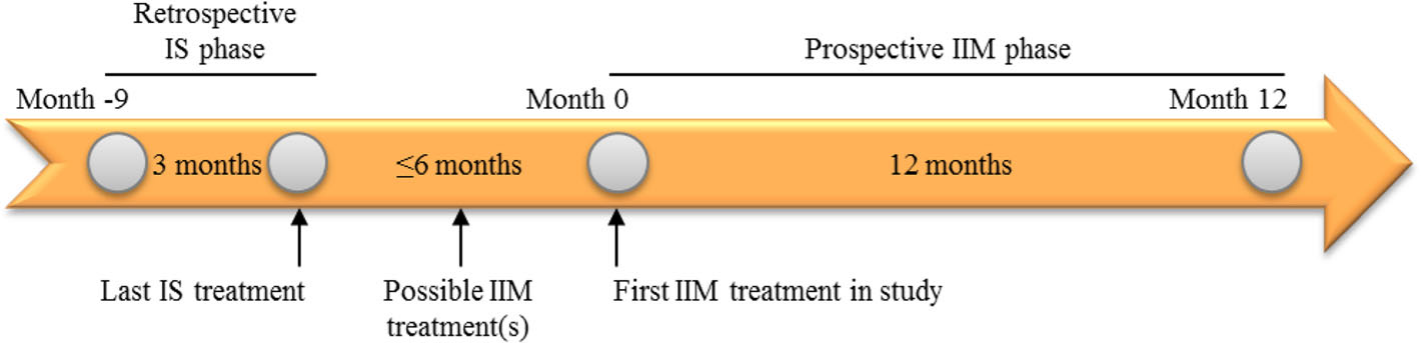
Study design. IIM = iron isomaltoside; IS = iron sucrose

This was a prospective, observational, multicentre study conducted at five clinical sites across Sweden and the United Kingdom (UK) between September 2014 and December 2016. Patients included were ≥ 18 years of age and in a stable phase of CKD, had been on HD therapy > 3 months, and had received at least one dose of IS treatment within the last 6 months before study start while being on HD. Exclusion criteria were IIM contraindications, inability to give informed consent, primary disease other than CKD and likely to impact the study results, inability to estimate retrospective data for IS treatment, and planned change of iron dosing protocol or routines during the study. The study design is shown in Fig. 1. Patients entering the study had to have retrospective 3-month data for IS therapy within month − 9 to month 0 from the start of the prospective phase. The IS that had been used was Venofer® (study centres personal communications). IIM was given as standard treatment according to the product label and local clinical practice, and was generally administered undiluted (50 mg/mL solution) as a fast-push injection into the venous limb of the dialyser. Most patients were observed for 12 months after the first treatment. All patients were treated and followed according to the local guidelines in each centre. At each participating centre, a senior nephrologist was responsible for the conduct of the study.

### Data collection and outcome measures

Data for iron treatment routine and dosing, blood tests, concomitant anaemia medications (ESAs, blood transfusion, and cobalamin and/or folic acid), and adverse drug reactions (ADRs) were collected from the medical records. The data collection was done prospectively for the IIM study phase and retrospectively for the historic IS phase. All blood tests were analysed locally at each participating centre. ADRs during the prospective IIM study phase were registered and reported in accordance with the national reporting systems. ADRs registered in the IIM study were also reported to the Sponsor’s pharmacovigilance department. For ADRs during the IS phase, only recorded events of metallic taste were collected. The collected data were systematically entered into an electronic case report form (eClinicalOS, Merge Healthcare, NC, USA; licensed by BioStata ApS, Denmark).

The primary endpoint was to demonstrate non-inferiority for maintenance of Hb levels over the first three months of IIM treatment compared to the historic 3-month phase with IS. Key secondary endpoints included assessments of iron and ESA dosing, laboratory parameter changes and maintenance, and frequency of ADRs.

### Statistical methods

Data analyses were conducted on the safety analysis set (*n* = 198), which included all patients who were enrolled in the study according to the protocol criteria and who received at least one dose of IIM, and on the effectiveness analysis set (*n* = 195), which included all patients in the safety analysis set who received at least one dose each of IIM and IS with at least one Hb measure post-treatment.

Data are presented as mean and standard deviation (SD) and as median and interquartile range (IQR) for continuous variables, and number of exposed patients (with proportions) for categorical variables. Data for IIM in 3-month intervals, 0–3 months, 3–6 months, 6–9 months and 9–12 months, of the prospective study were compared to cross-over retrospective data over 3 months for IS. For the primary endpoint analysis, non-inferiority for the mean Hb change for IIM treatment 0–3 months compared to the IS phase was determined using the 95% two-sided confidence interval (CI) and a margin of − 0.15 g/dL. Non-inferiority was declared if the lower limit of the 95% CI was above − 0.15. *P*-values for iron and ESA dosing and blood parameter levels were obtained from the Wilcoxon rank-sum test by comparisons of the IIM phases to the IS phase. P-values for comparisons of number of patients with metallic taste events between the IIM and IS phases were obtained from the McNemar test. The significance cut-off for all analyses was *p* < 0.05. All data were analysed using SAS version 9.4 (SAS institute, USA).

### Ethical considerations

The Regional Ethics Committees (RECs) in Sweden (EPN Lund; application number: 2014/306; approval date: 8th May 2014), and in the UK (East of Scotland Research Ethics Service; REC reference: 14/ES/1075; IRAS project ID: 159035; approval date: 23rd September 2014) approved the study. In the UK, local Research & Development permission was obtained from each participating centre prior to the start of the study. The study was registered with the ClinicalTrials.gov registry (NCT02301026). All study participants gave written informed consent before inclusion into the study, and the study was performed in accordance with the Declaration of Helsinki and the European Medicines Agency criteria for non-interventional studies [18].

## Results

### Study population

**Fig. 2:**
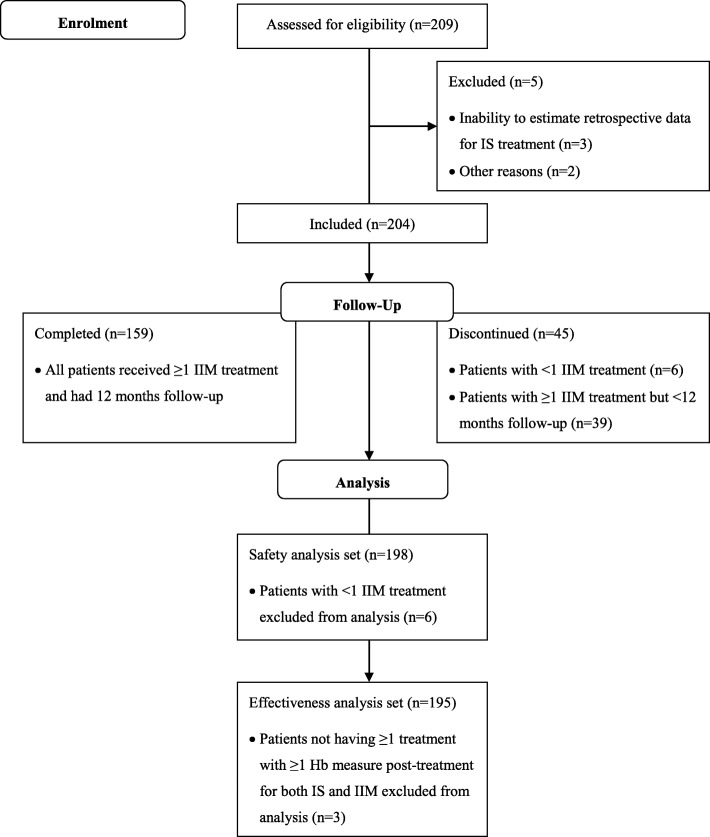
Patient flow diagram (based on CONSORT 2010). Hb = haemoglobin; IIM = iron isomaltoside; IS = iron sucrose

The details of patient disposition are outlined in Fig. 2. A total of 209 CKD patients on HD were screened for eligibility. Of the 204 patients enrolled, 159 (78%) patients completed the 12-month study and 45 (22%) patients discontinued. Patient demographics and baseline characteristics are presented in Table 1. The safety analysis set included 198 patients of whom the majority were male (*n* = 131; 66%), and 84% of the patients (*n* = 166) had received IIM treatment prior to study participation during a mean (SD) time of 3.8 (2.4) months from the last IS dose.

**Table 1:** Patient demographics and IV iron treatment characteristics at baseline

	Total population(*n* = 198)
Patient demographics	
Gender, n (%)	
Female	67 (33.8)
Male	131 (66.2)
Age (years)	
Mean (SD)	70.3 (13.0)
Weight (kg)	
Mean (SD)	78.2 (19.7)
IV iron treatment characteristics at baseline	
Time between last IS treatment and start of IIM prospective study phase^a^ (months)	
Mean (SD)	3.8 (2.4)
Patients with previous IIM treatment,^b^ n (%)	166 (83.8)

^a^Start of IIM prospective study phase determined by the date of informed consent. ^b^IIM treatment after the last IS treatment and before start of the IIM prospective study phase

*IIM* iron isomaltoside, *IS* iron sucrose, *IV* intravenous, *n* number of patients, *SD* standard deviation

### Iron treatment

The majority of patients (> 60%) followed a fixed low-dose iron treatment regimen across all 3-month periods of the IIM study and during the historic IS phase. IIM was generally administered undiluted as a fast-push injection with a mean (SD) time of around 1.0 (0.7) minute per treatment occasion. A mean (SD) time of around 2.9 (1.0) minutes were used for both preparation and intradialytic administration of IIM per treatment occasion. The mean number of iron doses and mean cumulative iron doses administered to patients did not differ significantly between the IIM and IS treatment phases (Table 2). Each patient received on average around 5 × 100 mg iron in each 3-month period. The mean (SD) cumulative iron dose monthly per patient was between 167.7 (89.1) and 187.0 (101.9) mg across all 3-month periods with IIM treatment, and was 178.1 (98.9) mg with IS treatment (Table 2).

**Table 2:** IV iron and ESA treatments

	Retrospective IS phase	Prospective IIM phases			
	0–3 months	0–3 months	3–6 months	6–9 months	9–12 months
IV iron treatment	*n* = 195	*n* = 195	*n* = 171	*n* = 156	*n* = 151
Number of IV iron doses per patient					
Mean (SD)	5.4 (3.0)	5.6 (3.1)	5.1 (2.8)	5.0 (2.7)	5.2 (3.0)
*p*-value^a^		0.4664	0.3533	0.2313	0.5874
Cumulative IV iron dose (mg) per patient					
Mean (SD)	534.4 (296.7)	561.0 (305.8)	515.2 (283.1)	503.2 (267.2)	528.5 (301.2)
Mean (SD) monthly	178.1 (98.9)	187.0 (101.9)	171.7 (94.4)	167.7 (89.1)	176.2 (100.4)
*p*-value^a^		0.3071	0.5650	0.3530	0.8036
ESA treatment	*n* = 195	*n* = 195	*n* = 171	*n* = 156	*n* = 151
Patients with ESA, n (%)	175 (89.7)	163 (83.6)	153 (89.5)	143 (91.7)	140 (92.7)
Number of ESA doses per patient					
Mean (SD)	19.4 (11.1)	17.5 (11.7)	20.1 (12.3)	18.6 (11.3)	20.1 (12.5)
Median (IQR)^b^	14.0 (12.0–26.0)	14.0 (8.0–25.0)	17.0 (10.0–29.0)	15.0 (9.0–26.0)	16.0 (9.0–32.0)
*p*-value^a^		0.0468	0.9228	0.2840	0.8250
Cumulative ESA dose (1000 IU) weekly per patient					
Mean (SD)	8.0 (8.1)	7.4 (6.5)	8.7 (8.0)	9.0 (9.3)	8.9 (7.4)
Median (IQR)^b^	6.0 (3.3–10.7)	5.7 (2.7–10.0)	6.7 (2.9–12.0)	6.0 (3.0–10.8)	7.0 (3.0–11.0)
*p*-value^a^		0.4131	0.5745	0.8107	0.3248

^a^*P*-value obtained from Wilcoxon rank-sum test by comparison to the retrospective IS phase

^b^Data not normally distributed are presented as median values

*ESA* erythropoiesis-stimulating agent, *IIM* iron isomaltoside, *IQR* interquartile range, *IS* iron sucrose, *IU* international unit, *IV* intravenous, *n* number of patients, *SD* standard deviation

### Concomitant anaemia medication

Concomitant medications for anaemia correction during the IIM and IS phases were similar and included ESAs (> 80% of patients across all 3-month periods; Table 2), cobalamin and/or folic acid (around 50% of patients across all 3-month periods), and blood transfusion (3–6% of patients across all 3-month periods).

For patients treated with ESAs, the mean number of doses given per patient over 3 months and the mean cumulative dose given per patient weekly did not differ significantly when comparing the IIM and IS treatment phases (Table 2).

### Effectiveness

Table 3 presents the mean levels and changes of Hb, ferritin, transferrin saturation (TSAT), and C-reactive protein (CRP) in each 3-month interval of the IIM and IS treatment phases. The effectiveness of IIM was comparable to that of IS with similar mean levels of Hb and iron parameters over the 3-month periods.

**Table 3:** Laboratory parameters

	Retrospective IS phase	Prospective IIM phases			
	0–3 months	0–3 months	3–6 months	6–9 months	9–12 months
Hb (g/dL)	*n* = 195	*n* = 195	*n* = 183	*n* = 178	*n* = 166
Hb level					
Mean (SD)	11.1 (1.0)	11.4 (0.9)	11.2 (1.0)	11.1 (1.1)	11.2 (0.9)
*p*-value^a^		0.0151	0.4154	0.8695	0.7934
Hb change vs. IS phase					
Mean (SD)		0.3 (1.2)	0.1 (1.3)	0.0 (1.4)	0.0 (1.2)
Ferritin (μg/L)					
Ferritin level	*n* = 187	*n* = 190	*n* = 182	*n* = 175	*n* = 159
Mean (SD)	395.5 (231.4)	395.1 (224.3)	420.4 (224.3)	408.4 (231.1)	420.8 (253.1)
Median (IQR)^b^	366.7 (227.5–519.0)	364.3 (222.7–523.0)	391.5 (261.3–553.3)	356.0 (236.7–549.0)	390.0 (223.0–590.0)
*p*-value^a^		0.9135	0.1799	0.5429	0.4434
Ferritin change vs. IS phase		*n* = 184	*n* = 175	*n* = 168	*n* = 152
Mean (SD)		4.2 (180.4)	28.4 (209.0)	9.6 (206.7)	16.8 (217.5)
Median (IQR)^b^		−20.3 (−93.7–93.4)	9.8 (−86.2–159.0)	−5.3 (−102–124.7)	4.7 (− 88.9–129.5)
TSAT (%)					
TSAT level	*n* = 187	*n* = 191	*n* = 182	*n* = 175	*n* = 159
Mean (SD)	25.0 (8.8)	26.8 (9.8)	26.4 (10.2)	26.2 (9.7)	26.7 (10.0)
p-value^a^		0.0592	0.4014	0.2828	0.1517
TSAT change vs. IS phase		*n* = 185	*n* = 175	*n* = 168	*n* = 152
Mean (SD)		1.8 (9.4)	1.0 (8.5)	0.9 (9.4)	1.3 (9.8)
CRP (mg/L)					
CRP level	*n* = 139	*n* = 129	*n* = 109	*n* = 122	*n* = 117
Mean (SD)	12.4 (14.8)	21.6 (35.1)	19.5 (25.5)	19.4 (27.9)	18.8 (23.6)
Median (IQR)^b^	7.0 (3.3–15.3)	8.7 (4.9–22.0)	10.0 (6.0–21.5)	10.0 (4.7–19.5)	10.0 (5.0–23.0)
*p*-value^a^		0.0351	0.0030	0.0143	0.0131
CRP change vs. IS phase		*n* = 124	*n* = 104	*n* = 112	*n* = 108
Mean (SD)		8.9 (33.9)	6.8 (21.8)	6.3 (19.9)	7.2 (21.6)
Median (IQR)^b^		0.8 (−1.5–7.0)	2.6 (−0.8–7.8)	1.6 (−1.1–10.8)	1.4 (−1.5–11.2)

^a^*P*-value obtained from Wilcoxon rank-sum test by comparison to the retrospective IS phase

^b^Data not normally distributed are presented as median values

*CRP* C-reactive protein, *Hb* haemoglobin, *IIM* iron isomaltoside, *IQR* interquartile range, *IS* iron sucrose, *IU* international unit, *IV* intravenous, *n* number of patients, *SD* standard deviation, *TSAT* transferrin saturation

**Fig. 3:**
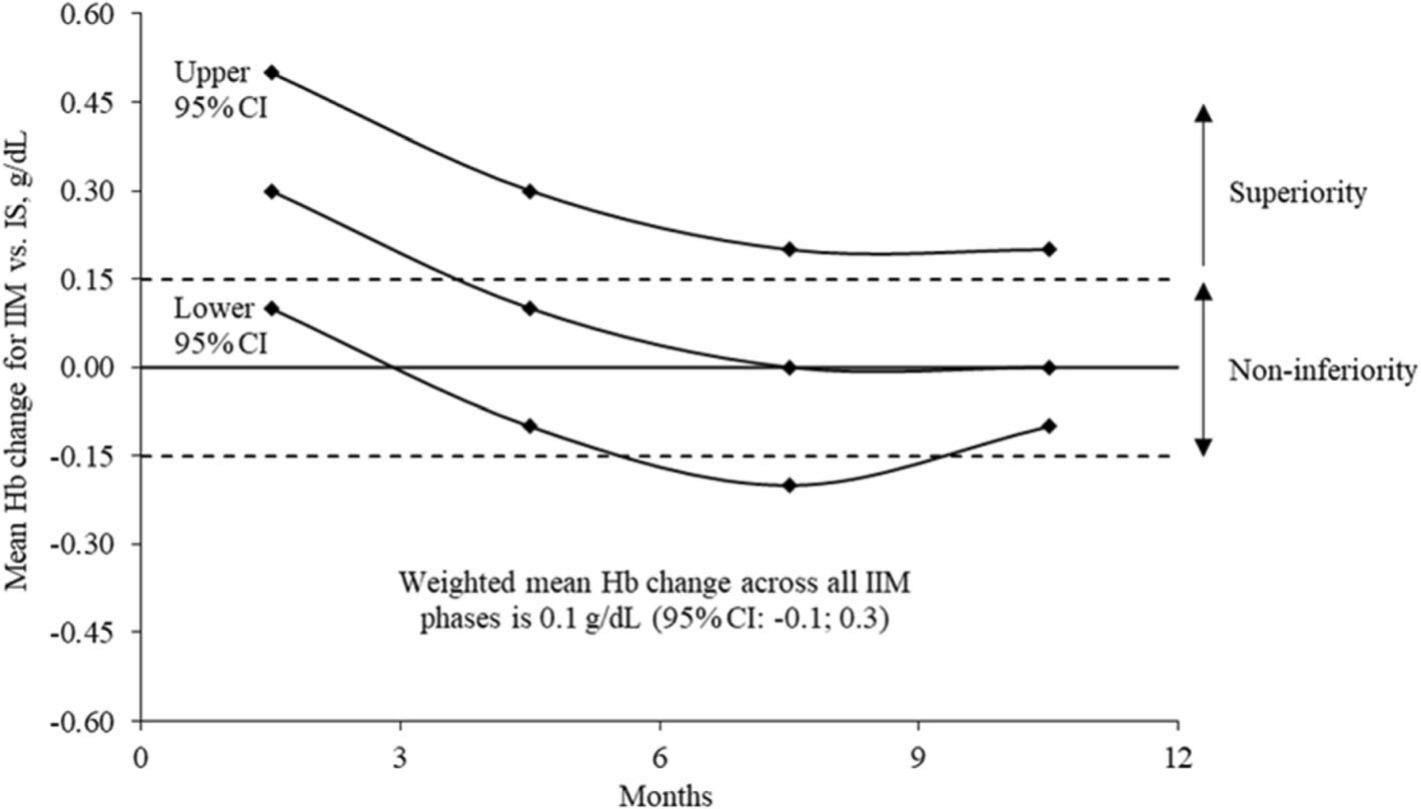
Mean Hb changes for the IIM prospective phases versus the IS retrospective phase. Non-inferiority was achieved for the primary endpoint comparison of IIM 0–3 months to IS where the lower limit of the 95% CI was above the margin of − 0.15 g/dL. CI = confidence interval; Hb = haemoglobin; IIM = iron isomaltoside; IS = iron sucrose

**Fig. 4:**
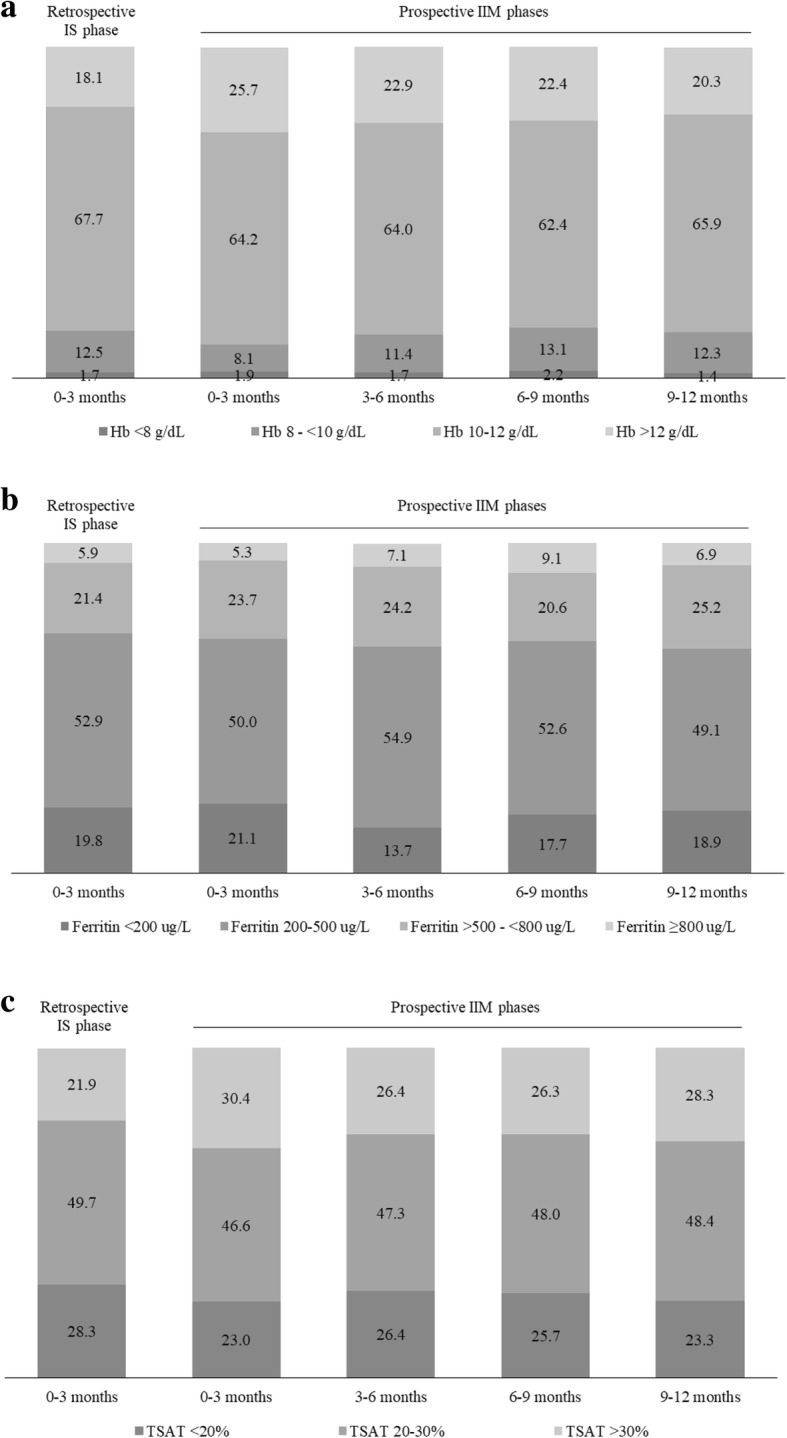
Percent distribution of all Hb values across Hb ranges (**a**), percent patient distribution across ferritin ranges based on individual mean values (**b**), and percent patient distribution across TSAT ranges based on individual mean values (**c**). The maintenance of Hb and iron parameters with IIM compared to IS treatment was similar. Hb = haemoglobin; IIM = iron isomaltoside; IS = iron sucrose; TSAT = transferrin saturation

The mean Hb level was around 11 g/dL for both iron drug phases. The primary endpoint analysis demonstrated that IIM was non-inferior to IS in maintenance of Hb levels. The mean (SD) change in Hb was 0.3 (1.2) g/dL (95% CI: 0.1, 0.5) when comparing the IIM 0–3 month period to the IS phase (Table 3 and Fig. 3). Non-inferiority to IS in Hb maintenance was also achieved for the IIM 3–6 and 9–12 month periods (Fig. 3). The percent distribution of all Hb values across different Hb ranges in a 3-month interval was similar between the IIM 3-month periods and the IS phase (Fig. 4a), and was based on 526 Hb measures for IS, 579 Hb measures for IIM 0–3 months, 536 Hb measures for IIM 3–6 months, 510 Hb measures for IIM 6–9 months, and 487 Hb measures for IIM 9–12 months. Most Hb values (62–68%) were maintained within the target range of 10–12 g/dL (including both values) with both iron treatments, and around 13% of the Hb values were < 10 g/dL. The maintenance of iron parameters with IIM compared to IS treatment was also similar. Approximately half of the patients had mean ferritin and TSAT levels within the desired ranges of 200–500 μg/L and 20–30% respectively (including both values), with both iron treatments (Fig. 4b and c). Around 30% of the patients had a mean ferritin level > 500 μg/L, but for most of these patients the mean ferritin level did not reach 800 μg/L (Fig. 4b). Between 20 and 30% of the patients had a mean TSAT level > 30% (Fig. 4c), and the maximum level across the 3-month periods was around 70% (Data on file, Pharmacosmos A/S, Denmark).

The median (IQR) level of CRP was slightly elevated during the IIM phase compared to the IS phase (9 [5–22] mg/L for IIM 0–3 months, 10 [6–22] mg/L for IIM 3–6 months, 10 [5–20] mg/L for IIM 6–9 months, and 10 [5–23] mg/L for IIM 9–12 months versus 7 [3–15] mg/L for the IS phase, *p* < 0.05; Table 3). Sub-analyses of CRP levels for each individual site in the study demonstrated that two of the five centres, in particular one of them, contributed to the observed increase in CRP between the IIM and IS phases (Data on file, Pharmacosmos A/S, Denmark).

### Safety

A total of 9 ADRs were reported in 4/198 (2%) of the patients who were treated with IIM. Two of these ADRs were reported as serious (burning sensation in the face, pruritus, back pain and vomiting in one patient, and upper abdominal pain, chills, pyrexia and nausea in another patient), and the rest were reported as not serious (headache in one patient, and 6 events of metallic taste in another patient). All patients had an uneventful recovery. The two patients who experienced a serious ADR discontinued the study.

The 6 events of metallic taste with IIM treatment reported in a single patient occurred out of a total of 3528 IIM administrations (0.2%) in the study. Five of these events took place during the first three months of IIM treatment and one event took place during the 6–9 month period of the study. The single patient who experienced metallic taste with IIM treatment had a history of frequent metallic taste events when administered IS (study nurse personal communication). The frequency of patients reporting metallic taste was significantly higher when treated with IS during the historic phase compared to when treated with IIM during the prospective study phase (34% [67/198] versus 0.5% [1/198], *p* < 0.0001; Table 4).

**Table 4:** Frequency of metallic taste

	Retrospective IS phase	Prospective IIM phases			
	0–3 months	0–3 months	3–6 months	6–9 months	9–12 months
	*n* = 198	*n* = 198	*n* = 172	*n* = 157	*n* = 152
Patients with metallic taste, n (%)	67 (33.8)	1^a^ (0.5)	0 (0.0)	1^a^ (0.6)	0 (0.0)
p-value^b^		< 0.0001	NA	< 0.0001	NA

^a^Same patient

^b^*P*-value obtained from McNemar test by comparison to the retrospective IS phase

*IIM* iron isomaltoside, *IS* iron sucrose, *NA* not applicable, *n* number of patients

## Discussion

This prospective observational study of IIM treatment in clinical practice demonstrates effective maintenance of Hb levels and adequate iron status with a good safety profile in CKD patients on HD converted from IS. When comparing IIM and IS therapies, both iron preparations showed similar effectiveness in maintaining Hb and iron parameter concentrations in the patients, with similar doses and ESA requirements. The primary endpoint was achieved and IIM demonstrated non-inferiority to IS in maintenance of Hb levels in the patients.

The treatment frequency and cumulative iron doses administered to the patients were comparable between the IIM and IS phases, and mainly followed a fixed low-dose treatment regimen given at regular intervals. Clinical guidelines recommend a low-dose high-frequency IV iron treatment protocol for adult HD patients who are iron deficient and receive ESA therapy [2]. For drugs given frequently it is advantageous to have a short duration of time for the handling and administration. The time for both preparation and injection of IIM into the dialysis circuit was on average 3 min. Although not assessed in this study, the ability to rapidly prepare and administer IIM as a fast-push injection is anticipated to enable logistical benefits in clinical practice, and this is likely to translate into efficiencies for HD departments using this drug.

The pattern of ESA use did not change over time in the IIM study or when compared to the IS phase probably because the iron protocol remained the same. This shows that ESA and IV iron were tightly co-medicated for the treatment of anaemia in this HD cohort. IIM and IS were equally effective in keeping the Hb levels, and the Hb values were generally within the target range of 10–12 g/dL during both iron treatment phases. A proportion of the Hb values were, however, below 10 g/dL with both iron treatments, which indicates suboptimal management of the anaemia for some of the patients.

When correcting anaemia with iron supplementation in adult CKD patients on ESA therapy, it is recommended to keep the ferritin level ≤ 500 μg/L [1] or ≤ 800 μg/L with a review of the iron dose when the ferritin level reaches 500 μg/L [2]. About 50% of the patients in this study maintained a mean ferritin level between 200 and 500 μg/L, and to a lesser extent between 500 and 800 μg/L, while a small percentage (< 10%) of the patients had a mean ferritin level ≥ 800 μg/L during the IIM and IS treatment phases. The high level of ferritin in these patients may reflect active inflammation or may have resulted from blood transfusions given to patients who had a very low level of Hb. High levels of ferritin in HD patients have been reported previously. In the 1-year Dialysis Outcomes and Practice Patterns Study (DOPPS) Practice Monitor conducted between 2010 and 2011, data for anaemia care collected from up to 120 HD facilities in the United States showed that the ferritin levels exceeded 800 μg/L in at least half of the patients in over a quarter of these facilities [19]. By 2011, the serum ferritin concentration was ≥800 μg/L in 34% of the patients, and was > 1200 μg/L in 11% of the patients.

Ferritin is commonly used as a marker to monitor the iron status in patients, but as an acute phase reactant its blood level increases in systemic inflammation independently of body iron stores. Non-invasive techniques, such as quantitative magnetic resonance imaging (MRI), provide a more direct and accurate measure of body iron stores [4]. In previous MRI studies, the liver iron concentration in HD patients treated with IV iron was found to correlate with the administered iron dose, but inconsistently with the serum ferritin level [20–22]. Furthermore, hepatic iron overload detected with MRI was reported in a French HD cohort of 119 patients when treated with IS according to current guideline recommendations (ferritin target range 200–500 μg/L) [22]. In this HD cohort, 84% of the patients had a liver iron concentration corresponding to mild, moderate or severe hepatic iron overload (cut-off > 50 μmol/g dry weight).

Iatrogenic iron overload longer term may be associated with an increased risk of cardiovascular events and mortality in HD patients [4]. This has led to questioning of the safety of contemporary IV iron dosing protocols in the HD setting. In the current study, the mean cumulative monthly doses of IIM ranged from 168 to 187 mg, and the majority of the Hb values were maintained within the target range of 10–12 g/dL. This iron dosing is rather modest in relation to the UK guideline recommendation for iron maintenance therapy in HD patients of 50–60 mg IV iron per week (200–240 mg monthly) [2]. It is, however, higher than the very conservative strategy advocated by the Japanese guidelines of 40 mg IV iron per week (160 mg monthly) for HD patients in a cycle of 13 administrations, while keeping the serum ferritin level < 300 μg/L [23]. From a safety perspective, the mean cumulative monthly doses of IIM administered to the patients in this study to maintain a stable Hb are below IV iron dose levels of ≥200 or ≥ 300 mg per month that have been found in recent long-term (1–2 years) epidemiological studies to be associated with an increased risk of morbidity and mortality in HD patients [24, 25]. Furthermore, IS doses of > 250 mg per month have been reported to be associated with an increased risk of hepatic iron overload, as determined with MRI in a French study in 199 HD patients [26].

The CRP levels in the present study were found to be slightly higher during the IIM treatment phase compared to the IS treatment phase. Sub-analyses revealed that the difference in CRP levels between the iron drug phases was, however, only observed for two of the five participating centres, and the centre for which this difference was most evident had the largest number of patients in the study. Thus, this effect was mainly derived from one centre that also had the greatest contribution of patients in the study. The reason for this trend is unclear but may have been attributed to concomitant diseases or the dialysis process per se. It is unlikely to have resulted from the iron treatment because the iron dosing and levels of iron parameters were similar between the IIM and IS phases.

In this 12-month study, IIM demonstrated a good safety profile, consistent with the findings from other clinical studies of IIM in CKD patients on dialysis [5, 9, 14, 27]. Only one patient experienced metallic taste during the IIM treatment phase. The patient reported metallic taste at several occasions, but had a known history of frequent metallic taste events when administered IS. In this study cohort, IIM was associated with a significantly reduced frequency of metallic taste sensation compared to IS, which is likely to be desirable to patients. In a previous randomised trial comparing the treatment effects of IIM and IS in patients with iron deficiency anaemia, the frequency of patients reporting dysgeusia that mainly consisted of metallic taste (Data on file, Pharmacosmos A/S, Denmark) was lower in the IIM group (0.6%) than in the IS group (2.4%) [28], providing further evidence for a difference in taste sensation between the two iron drugs. The impact of the difference in metallic taste frequency between IIM and IS in the present study is unknown. The metallic taste could turn out to be a surrogate indicator for labile iron release, but this is unproven and requires investigation.

The tight binding of iron to the carbohydrate moiety in IIM, allows a controlled and slow release of iron to iron-binding proteins, which may have potential benefit to minimise labile iron toxicity. Less stable IV iron preparations are at increased risk of triggering toxicity such as oxidative stress, renal injury, and endothelial damage, as demonstrated in studies with CKD patients treated with IS [29, 30]. Iron-induced oxidative stress and endothelial dysfunction may promote atherosclerosis with increased risks of cardiovascular morbidity and mortality in HD patients [31]. There is, however, overall conflicting data for an association between IV iron treatment and elevated risk of cardiovascular adverse outcomes due to oxidative stress in HD patients [32].

Another area of controversy is the concern of increased infection risk with IV iron therapy caused by enhanced bacterial growth and altered host immunity [33]. While some studies have reported a link of usage, dose-dependent risk or frequency-dependent risk between IV iron and infection or infection-related fatalities in CKD patients, other studies have not confirmed such a link [32]. A study in dialysis patients comparing different IV irons found that less stable preparations such as IS have an immunomodulatory effect leading to reduced macrophage function in contrast to more stable preparations, such as IIM, that do not show this effect [34]. The clinical relevance of this finding on infectious complications in patients on dialysis receiving repeated administrations of less stable IV iron formulations remains to be explored.

In this study, each patient served as its own comparator for the IIM vs IS treatment. The use of a historic treatment phase can be subject to confounding due to time effects. However, since the patients were in a stable phase of the disease and the duration of the study was relatively short, the use of such a historic treatment phase seemed reasonable. For each patient, the analysis was based on data aggregated in 3-month periods, where the average of all measurements in each period was used. Such an approach may cause regression of values to the mean when assessing the average and range out of averages for a population, but was deemed feasible for this study given that the patients were on stable HD and anaemia management and thus no large intra-individual variability in the data across 3 months was expected. Data for ADRs reported during the historic IS treatment phase were generally considered difficult to access and follow up. The comparison of the safety profiles for IIM and IS was therefore limited to “yes or no” data for patients experiencing metallic taste to minimise the risk of inaccuracies for the retrospective IS data collection.

## Conclusions

In conclusion, this study shows that IIM is as effective as IS in managing iron deficiency anaemia in CKD patients on HD, and that it has a good safety profile. The fast-push injection of IIM is predicted to enable logistical benefits in clinical practice, and the low frequency of metallic taste events further contributes to the patient convenience.

## Abbreviation

ADR: adverse drug reaction
CI: confidence interval
CKD: chronic kidney disease
CRP: c-reactive protein
DINO: DIafer NOn-interventional
DOPPS: Dialysis Outcomes and Practice Patterns Study
ESA: erythropoiesis-stimulating agent
Hb: haemoglobin
HD: haemodialysis
IIM: iron isomaltoside
IQR: interquartile range
IS: iron sucrose
IU: international unit
IV: intravenous
MRI: magnetic resonance imaging
n: number of patients
NA: not applicable
PIVOTAL: Proactive IV Iron Therapy in Dialysis Patients
REC: Regional Ethics Committee
SD: standard deviation
TSAT: transferrin saturation
UK: United Kingdom
